# A system for in-situ, wave-by-wave measurements of the speed and volume of coastal overtopping

**DOI:** 10.1038/s44172-023-00058-3

**Published:** 2023-03-03

**Authors:** Margaret J. Yelland, Jennifer M. Brown, Christopher L. Cardwell, David S. Jones, Robin W. Pascal, Richard Pinnell, Tim Pullen, Eunice Silva

**Affiliations:** 1 National Oceanography Centre, European Way, Southampton, SO14 3ZH UK; 2 National Oceanography Centre, 6 Brownlow Street, Liverpool, L3 5AD UK; 3 HR Wallingford, Howbery Park, Wallingford, Oxfordshire OX10 8BA UK

**Keywords:** Natural hazards, Environmental sciences

## Abstract

Wave overtopping of sea defences poses a hazard to people and infrastructure. Rising sea levels and limited resources mean accurate prediction tools are needed to deliver cost-effective shoreline management plans. A dearth of in-situ data means that the numerical tools used for flood forecasting and coastal scheme design are based largely on data from idealised flume studies, and the resulting overtopping predictions may have orders of magnitude uncertainty for complicated structures and some environmental conditions. Furthermore, such studies usually only provide data on the total volume of overtopping water, and no data on the speed of the water. Here we present WireWall, an array of capacitance-based sensors which measure the speed and volume of overtopping water on a wave-by-wave basis. We describe the successful validation of WireWall against traditional flume methods and present results from the first trial deployments at a sea wall in the UK. WireWall results are also compared with numerical predictions based on EurOtop guidance. WireWall technology offers an approach for reliable acquisition of the data needed to develop resilient coastal protections schemes.

## Abstract (web summary)

Numerical tools for flood forecasting and for designing coastal protection schemes require accurate real world data on speed and volume of overtopping waves on sea walls. Margaret Yelland and colleagues here describe the validation and field deployment of arrays of capacitance sensors, termed Wirewall, as a tool for acquisition of detailed data of coastal overtopping.

## Introduction

Countries that have a coastal border often have many kilometres of sea defences to protect people, property, transport networks and critical infrastructure from hazardous wave overtopping and flooding. The UK, for example, has over 3000 kilometres of coastal defences and the cost of building a sea wall is typically £10,000 to £25,000 per linear meter. Numerical predictions of wave overtopping are used for: designing new or replacement sea defences; planning maintenance works; forecasting flood hazard; and delivering safe operation of services. However, overtopping predictions from widely-used industry guidance (EurOtop^1^) and calculation tools (e.g. Bayonet GPE^2^) currently may have orders of magnitude uncertainties^1^ due to the limitations of the dataset (CLASH^3^) on which they are based: some structures (e.g. some types of defence common in the UK) and conditions are better represented in the database than others.

In-situ overtopping measurements are very scarce and have mostly been obtained on grassy^4^ and concrete^5^ dikes and rubble breakwaters:^6^ these data are not applicable to other structures such as vertical sea walls^7^. Due to this lack of field data, numerical tools are largely based on^8^ or heavily supplemented by^9^ measurements made during idealised, 2-dimensional studies in wave flumes. Such studies are also limited in that they: generally focus on the more common, simple sea wall designs (e.g. vertical walls with no stepped revetment); assume conditions are uniform across the flume (i.e. alongshore); use a static beach profile and a particular range of water level and wave conditions (which may exclude conditions such as lower water levels which generate modest overtopping rates); do not include the influence of wind; poorly represent effects such as spray which cannot be scaled. In contrast, the profile of a real-world sea defence can vary along its length, and beach levels can change both slowly due to seasonal effects and rapidly due to the impact of storms^10^. In addition, wind can be an important factor^11^ since it can drive water inland that would otherwise have been reflected back to sea due to the presence of a return curve on a sea wall.

Traditional flume studies generally use collection tanks to determine the total volume of water that overtops a physical model of a particular sea defence during an event that is represented by a single combination of water level, beach profile and wave conditions. Data on the overtopping volumes from individual waves are not usually obtained, i.e. the number and magnitude of individual wave events cannot be determined. In addition, the speed of the overtopping water is very rarely measured (in particular for dense spray overtopping) even though speed is a critical factor when estimating hazard, particularly to pedestrians^12^. Other methods such as optical techniques^13^ and resistance gauges^14^ have occasionally been trialled in flume studies of green water overtopping (rather than spray) but these techniques have not been scaled up for field deployment. Radar^15^, laser^16^ and (camera) image-processing^17,18^ technologies are also being investigated as potential ways to measure overtopping in the field but these approaches are subject to weather conditions (affecting the signal intensity), blockage (e.g. a person standing in front of the scan/within the image) and the assumptions made to calibrate them^5^. Again these techniques are generally limited to green water and require calibration/validation against other methods, such as collection tanks^15^. However, collection tanks in the field are either embedded within a structure^19^ or are temporary freestanding installations. The latter are too cumbersome to deploy regularly, often only being in place for short periods (typically a month^20^), are unsuited for deployment on many sea walls, and may themselves pose a hazard if they are unable to withstand severe events. In some locations, photographic images obtained through social media or new citizen science initiatives could potentially be used to derive information about the occurrence of overtopping^21^, but this information is qualitative rather than quantitative and would often miss events, particularly at night.

Traditional flume studies are often focussed on extreme, relatively infrequent, conditions^20^, and the resulting uncertainties in the numerical predictions for these conditions are currently estimated as a factor of 3. Fewer studies have focussed on the more frequently occurring, less energetic (nuisance) type of overtopping that can nevertheless pose a hazard to pedestrians^21^, as well as contribute to flood events. The numerical predictions for these less energetic conditions can have much larger uncertainties, rising in some cases to orders of magnitudes particularly if the structure in question is a complicated one, or is not well represented in the underlying database. All uncertainties have cost (and carbon) implications for engineering works, and can severely degrade the accuracy of hazard alert services due to missed, or false, warnings.

With rising sea levels, hazardous wave overtopping events are increasing in frequency, intensity and duration^22^ and coastal management plans have to take these future climate change impacts into consideration. The high cost of sea defences along with limited resources mean that more accurate prediction tools are urgently needed to deliver cost-effective shoreline management plans. More accurate tools require data from field measurements of coastal overtopping. New measurement systems are therefore needed that are capable of providing quantitative data continuously for extended deployments of months or years.

In this paper we present a novel measurement system WireWall that can measure the speed and volume of overtopping water (spray as well as green water) on a wave-by-wave basis, and is designed to be deployable long-term on a very wide range of coastal defence types. We describe the successful validation of WireWall against traditional collection tanks during flume experiments, and present results from the first short (single tide) field deployments of a prototype system at Crosby, on the northwest coast of England (see Methods section for a description of the site, and photographs of the sea wall). Comparisons with numerical predictions from Bayonet GPE are also discussed.

## Results

WireWall is based on capacitance-wire technology previously developed for measuring breaking waves in the open ocean^23^. Details of the WireWall system can be found in the methods section and the WireWall project report^24^ and are only summarised here. The system contains multiple capacitance sensors, each consisting of a PTFE coated wire and an uninsulated electrical return wire spaced about 1 cm apart. When green water or dense spray forms a bridge (or multiple bridges) between the two wires, a signal is produced that is proportional to the cumulative length of the wetted sections of wire. The signal contains no information about the location of the water on the wire, e.g. a body of water 10 cm tall hitting the sensor would produce the same signal regardless of whether it impacted the upper or lower part of the sensor. Two such bodies simultaneously impacting different parts of the sensor would produce the same signal as a single 20 cm body of water. Multiple sensors are mounted vertically and arranged in a row from the crest of the structure to some distance inland. The time delay between water arriving at one sensor and the next gives the speed of the flow in the direction of the row. The volume of water passing any individual sensor is calculated from the speed of the wave event, the duration of the event and the length of wetted wire during the event. See Methods section for details.

In the flume studies and field deployments described below, multiple WireWall units were deployed side-by-side, with each unit containing 6 sensors in a sea-to-land row. This provided multiple estimates of speed and volumes to be obtained for each individual wave event, which could be used to assess measurement uncertainty and/or real variability in the results. It also allowed the sea-land distribution of overtopping volumes to be determined. For the field deployment, use of multiple units with 6 sensors each also provided redundancy in case of damage during violent events.

### Choice of site and design of the tests

The location chosen for the initial field tests of the system was at Crosby, on the northwest coast of the UK. This site was chosen for (a) its proximity to the National Oceanography Centre’s laboratories in Liverpool, which made it possible to deploy the system at quite short notice when the weather and wave forecasts indicated that overtopping was likely^21^, (b) the availability of existing wave, water level, and meteorological monitoring stations, (c) various logistical considerations such as obtaining permission to install the system (using existing contacts within local authorities) and access, (d) the interest of other local stakeholders such as the local Environment Agency. The system itself was designed to be flexible, e.g. the separation of the sensors in the sea-land direction could be made smaller so that slow, less energetic wave events would be detected on all sensors, or larger if faster, high-energy wave events were expected so that uncertainties in the speed and volume measurements could be minimised.

Once the field site was chosen the initial validation tests in the flume were designed to represent the structure at Crosby, and the wave and water level conditions that might be encountered there. The parameter ranges tested in the flume were chosen using historical, qualitative observations of overtopping at Crosby along with Bayonet GPE predictions of the overtopping derived from the wave and water level conditions that were associated with those observations^21^. During the field trials at Crosby^25^, wave and water levels during the 2019 deployments were similar to the less energetic conditions simulated in the flume^26^ (WC12, 13 and 15 in Table 2, Methods).

The HR Wallingford team designed a collection tank with partitions spaced about 8 cm apart in the sea-land direction with the aim of collecting data on the inland distribution of overtopping water. The sensors in the WireWall system were also separated in the same fashion, with the aim of comparing the WireWall data directly with that from the tank partitions.

### Validation of speed measurements and tests against collection tanks in a flume

**Fig. 1:**
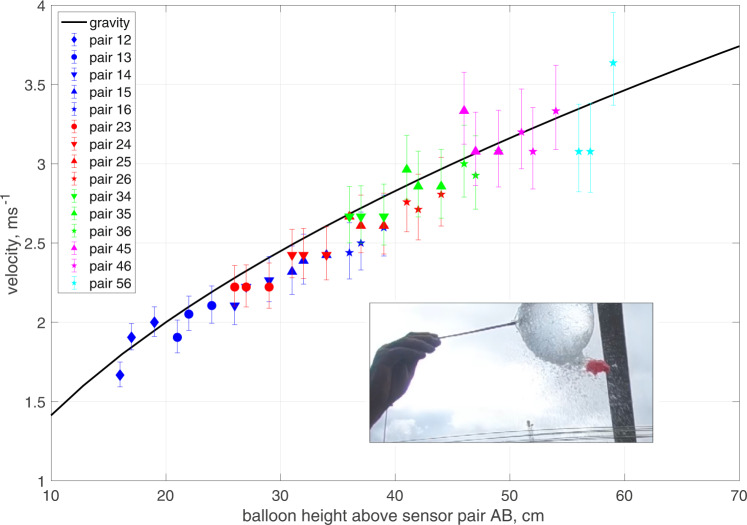
WireWall measurements of the speed of water falling from burst balloons (inset). The solid black line shows the expected speed from the acceleration due to gravity. Error bars represent + /−1 sample at a sampling rate of 400 Hz. Three balloon bursts at heights of 11 cm, 12 cm and 14 cm above the top sensor are shown. The legend indicates the various sensor pairs (AB) used for each estimate (dark blue symbols for pairs 1B, red for pairs 2B, green for 3B, pink for 4B and pale blue for 5B). The heights are measured from the bottom of the balloon to the mid-point between each pair. These data are available from the British Oceanographic Data Centre (10.5285/acd939f0-38e8-57b0-e053-6c86abc0aa19).

No independent measurement of the speed of the water flow was available, either in the flume tests or in the field trials. Instead, this aspect of the system was investigated during the initial design phase by arranging 6 sensors horizontally, one above the other, and bursting water-filled balloons at known heights above them. For each balloon test fifteen velocities were obtained^27^ by using every permutation of sensors pairs (sensor 1 was uppermost and 6 lowest) and the results were compared with the velocity expected from the acceleration due to gravity (Fig. 1). The agreement was very good, particularly since the sensors were only 10 cm apart. This close spacing means that any errors in determining the time of arrival of the water at a given sensor could produce relatively large errors in the calculated speed of the water, and hence the calculated volume. For example given the 400 Hz maximum sampling rate, if water was detected at sample n + /−1 rather than n (i.e. a time of arrival error of just 1/400 s), this would result in a measurement error of + /−0.3 m s^−1^ for a fall speed of 3.5 m s^−1^. The n + /−1 errors are shown in Fig. 1.

The prototype WireWall system was then trialled in a wave flume at HR Wallingford (see Methods for details). A 1:7.5 scale model of the sea wall (3.69 m tall) at Crosby was used: the vertical sea wall has a recurve at the top and a stepped revetment below (see Methods for image of the sea wall). The bathymetry used in the flume was derived from a survey at Crosby and the wave and water level conditions simulated those that might be expected to occur at the field site. Behind the sea wall were three long collection tanks arranged side-by-side. Two WireWall units were mounted just above, and slightly to either side of, the tanks (see photo in Methods). WireWall unit A transmitted a signal to the other unit (B) which allowed data from the two to be synchronized at the 400 Hz sampling frequency. Each unit carried 6 sensors: the first sensor was mounted at the crest of the wall; the second just inland of the wall above the seawards-most edge of the tanks, 7 cm inland of the first sensor; the remaining four sensors were spaced 10 cm apart progressively further inland. Additional WireWall sensors were also deployed inside the collection tanks to provide continuous (1 Hz) measurements of water depths. Prior to deployment in flume the WireWall sensors were calibrated by immersing them in known depths of water: no subsequent adjustments or corrections were made.

Various combinations of wave spectra and water level conditions were selected to represent those that might occur at Crosby^24^. In some tests each wave and water level combination was run for up to an hour in order to obtain at least 1000 waves so that the data were suitable for inclusion in the EurOtop database. For these longer runs pumps were used inside the tanks to prevent them overflowing. Corrections were applied to the data to allow for the flow rate of the pumps but since the pumps had to be turned on and off repeatedly during these tests this added additional uncertainty to the results from the tanks (see Methods): use of the pumps caused an uncertainty of about 20% in the tank data. In other tests multiple short runs (about 10 min) of a given wave and water level combination were performed to investigate other sources of uncertainty due to e.g. real variability in the overtopping results from the tanks and the variability of overtopping across the width of the flume. These runs had to be short in order to avoid the need to use pumps and the uncertainties that their use would have caused.

**Fig. 2:**
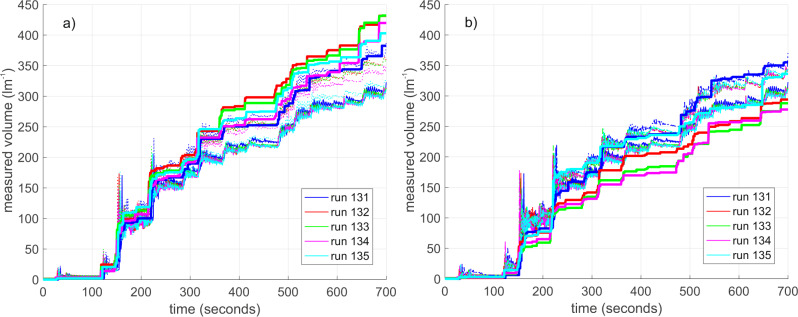
Measured wave-by-wave cumulative volumes (l m^−1^) during five short runs of a single wave and water level combination. The colours indicate the different runs as shown in the legend. **a**) results from: WireWall unit A (thick solid lines); tank C in the middle of the flume (thin solid lines); and tank A (dotted lines) which was offset to one side of the flume near to WireWall unit A. **b**) results from the WireWall unit B (thick sold lines); the same data from tank C in the middle of the flume (thin solid lines); and tank B (dotted lines) which was offset to the other side of the flume, near to WireWall unit B.

**Fig. 3:**
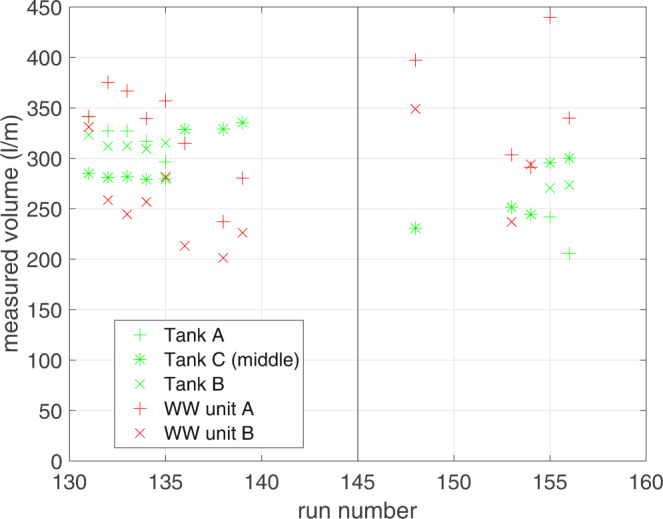
Measured volumes from repeated short runs in the flume. Tank C (green stars) was located centrally in the flume, with tanks A (green cross) and B (green x) offset to either side. The two WireWall (WW) units were offset further still, with WireWall unit A (red cross) at the far (wall) side of tank A and WireWall unit B (red x) at the far (wall) side of tank B. Two different water level and wave combinations are shown, with the vertical line dividing them. These data are available from the British Oceanographic Data Centre (10.5285/acd939f0-38e8-57b0-e053-6c86abc0aa19).

Figure 2 shows the wave-by-wave overtopping data^26^ from the collection tanks (without pumps) and from the WireWall systems, obtained during some of the short runs. It can be seen that the agreement between the WireWall units and the tank data is very good, but there is variability in the results, from both WireWall and the tanks, from one run to the next. This variability is least for tank C which was located centrally in the flume, and is larger for tanks A and B which were offset to either side of tank C. Cross-flume variability is also seen, with the volumes collected by the central tank C being smaller than those from the outer two tanks. However, this pattern was not consistently seen since in other runs tank C sometimes collected larger volumes than the outer two tanks (Fig. 3): such cross-flume variability may become less in the longer (~1000 wave) runs. In addition, variability from one run to the next was also seen in the tank C (which is assumed to be the least affected by wall effects). For example, eight runs (131 to 139, with one failed run) were made using a single wave and water level combination, but after the first five the flume was drained, to allow the outer two tanks to be moved further inland, and then refilled. It can be seen in Fig. 3 that the volumes in tank C were 33% greater in the last three runs compared with the first five. The variability in the WireWall results is slightly larger than that from the tanks, but this would be expected since (a) the WireWall sensors were located closer to the edges of the flume than were the tanks and (b) WireWall measures only a very thin slice of the chaotic overtopping water whereas the tanks were 10 cm wide. Results from the longer runs (below) showed less variability.

**Fig. 4:**
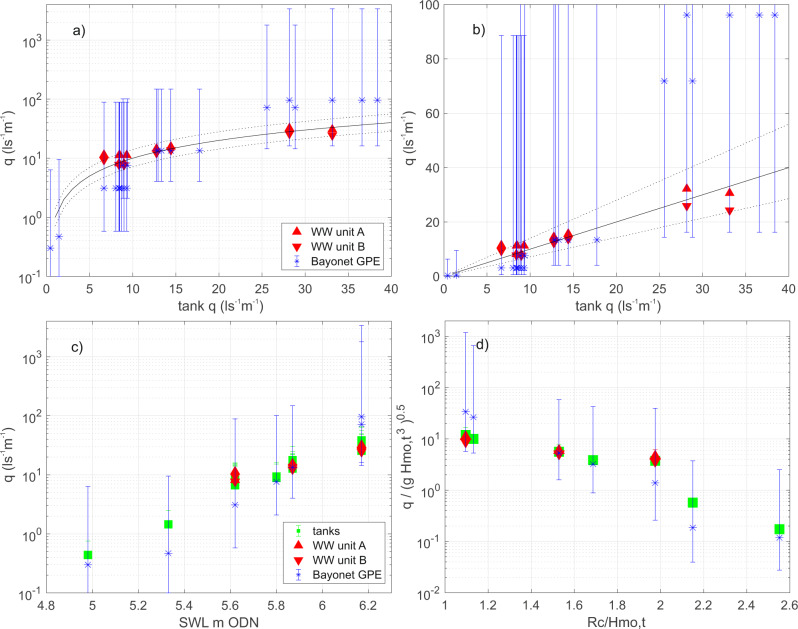
Comparison of total discharge rates. **a**) total discharge q (l s^−1^ m^−1^) data from the collection tanks on the *x*-axis, and from the two WireWall (WW) units (red triangles) for the long, 1000+ wave runs in the flume (see Table 2 in Methods). The results are scaled up to show real-world values. The black line indicates 1:1 agreement and the dotted lines indicate the + /−40% uncertainty estimated for the tank data. Also shown are the Bayonet GPE predictions (blue stars), with the error bars indicating + /−1 s.d. uncertainty. **b**) as **a**) but with a linear *y*-axis scale to show the tank and WireWall comparison in more detail. **c**) the same discharge data but displayed against real-world Still Water Level (SWL, m ODN) with the tank data shown as green squares. **d**) as **c**) but non-dimensionalised, where Rc is the freeboard (promenade height 7.2 m - SWL), Hmo,t is the significant wave height at the toe of the structure and g is acceleration due to gravity. Note that the WireWall systems were only deployed for some of the 1000+ wave runs. These data are available from the British Oceanographic Data Centre (10.5285/acd939f0-38e8-57b0-e053-6c86abc0aa19).

Figure 4a, b compares the WireWall measurements of total overtopping discharge q (l s^−1^ m^−1^, converted from the 1:7.5 flume scale up to real-world scale), with those of the collection tanks, from the long, ~1000 wave runs where pumps were used in the tanks to prevent them overflowing. The agreement is excellent, with WireWall results agreeing with the tank data to within the + /−40% uncertainty in the tank data. The uncertainty in the tank data was estimated from; multiple runs of the same conditions (to estimate uncertainty in the actual overtopping volumes), e.g. Figure 3; multiple tanks located side-by-side (to obtain lateral variability across the flume), e.g. Figure 3; and experiments to determine the errors in the correction used to allow for the use of pumps in the tanks (responsible for half the total uncertainty). The agreement between the two WireWall units was generally within about 10%, which is similar to the cross-flume uncertainty. Figure 4c shows the same data versus the real-world still water level (SWL, relative to Ordnance Datum Newlyn) and Fig. 4d the data after non-dimensionalising.

Estimates of total q were also obtained from the Bayonet GPE tool for each of the long flume runs. The + /−1 s.d. uncertainty estimates for the results from the Bayonet GPE tool encompassed the tank results. However, the mean Bayonet GPE predictions were biased low by a factor of 2 to 3 for some of the less energetic runs with smaller overtopping volumes, and biased high by a factor of 2 to 3 for the two most energetic runs (Table 2 in Methods). The biases in the mean Bayonet GPE predictions appear to be correlated with SWL.

### Field trials at Crosby: WireWall results and comparison with Bayonet GPE predictions

After the successful validation tests in the flume at HR Wallingford, a prototype WireWall system was trialled at the sea wall in Crosby, on the northwest coast of England where the 900 m long seawall is nearing the end of its design life. During winter 2018/2019 the system was deployed temporarily at the northern end of the sea wall during a number of spring tides when water levels were expected to exceed mean high water spring (MHWS 4.46 m OD).

The system used three WireWall units located side by side about 45 cm apart (see Methods). Each unit carried 6 capacitance sensors arranged in a row from sea to land, with a spacing of 35 cm between each sensor. The sensors were 2.4 m tall. Use of multiple units provided redundancy, which was particularly important for these early trials since the prototype system sometimes suffered from water ingress in the various connectors, or damage to the sensors (usually while the system was being installed). For each unit, the first sensor in each row projected seawards of the sea wall crest; the second was located at the crest; the third was 35 cm inland of the crest, just inland of the handrail that ran along the promenade; and the other three were further inland, with the 6th being about 1.4 m inland of the crest. The sea wall at Crosby has a recurve that deflects much of the water vertically upwards and offshore: this meant that a large fraction of the water detected on sensor 2 at the crest often returned directly back to the sea, i.e. the water did not propagate inland to the railing or beyond. For this reason we believe that the results from sensor 3 at the railing best represent the total overtopping on the promenade and also provide the best estimate of the hazard to pedestrians.

**Fig. 5:**
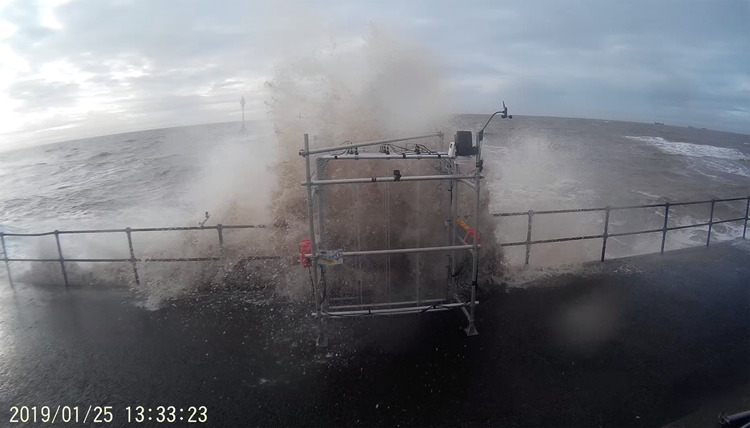
Image from 25th January 2019 showing one of the largest overtopping plumes observed during the deployment. The seawards part of the plume is taller than the rig. However, the upper part of the plume is made up of quite fine spray compared to the much denser spray/solid water lower down. In addition, the height of the plume reduces as it travels inland through the rig.

**Fig. 6:**
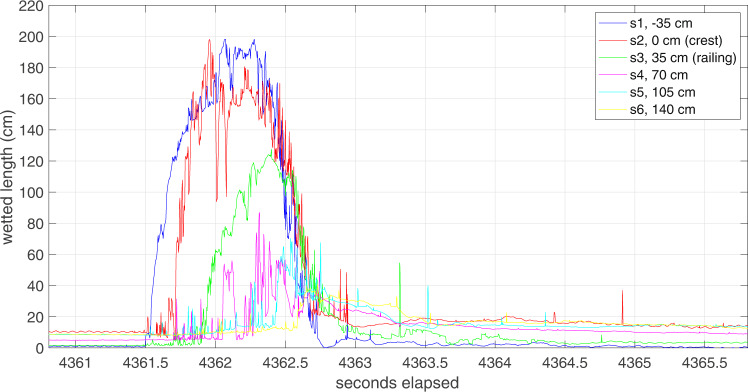
Data from one of the largest individual overtopping events on the 25th January 2019. A timeseries of wetted length data recorded at 400 Hz from the six sensors on WireWall unit A located at various distance inland from the crest: sensor 1 (s1, dark blue line) projected 35 cm seawards of the crest; sensor 2 (s2, red line) was at the crest; sensor 3 (s3, green line) was 35 cm inland of the crest immediately inland of the railing; sensor 4 (s4, pink line) was 70 cm inland of the crest; sensor 5 (s5, pale blue line) was 105 cm inland of the crest; sensor 6 (s6, yellow line) was 140 cm inland of the crest. The plot shows 5 s of data (seconds elapsed since logging started) and the event lasted for about 1.5 s.

Overtopping occurred during four out of the eight deployments carried out. The most energetic overtopping took place on the 25th January 2019 (Fig. 5). During this deployment only WireWall unit A obtained good data from all 6 sensors, but the other two units each had four sensors working (see notes in Table 1). Figure 6 shows the raw 400 Hz data from one of the largest individual events that day, and the propagation of the overtopping water from the seawards sensor to the inland sensor can be seen. One of the largest sources of uncertainty in the WireWall data is the speed of the flow (essential for calculating volume). To calculate the speed of the flow between pairs of sensors, an accurate time for the arrival of the event is needed: this can be difficult to determine since the detection of very small droplets arriving ahead of the main body of water makes the signal rather noisy. For this reason, for each event multiple speed estimates are made from every sensor that the event reaches: if the event reaches all 6 sensors this provides fifteen estimates of the speed. For each event, the median of those speeds is then applied to the measured depth on each sensor to calculate the volume passing that sensor.

**Table 1:** Results for the eight deployments during the field trials at Crosby.

Deployment			Bayonet GPE volumes (m^3^ m^−1^) +/− uncertainty.					WireWall volumes (m^3^ m^−1^) on different sensors.					
num.	date 2018/19	HW, GMT	−2 s.d.	−1 s.d.	total	+1 s.d.	+2 s.d.	sensor 2 crest	“sensible” crest	sensor 3, 35 cm	sensor 4, 70 cm	sensor 5, 105 cm	sensor 6, 140 cm
1	Oct 24	10:42			0.00			0.0	0.0	0.0	0.0	0.0	0.0
2	Oct 25	11:15	0.00	0.00	**0.01**	0.10	1.0	0.0	0.0	0.0	0.0	0.0	0.0
3	Oct 26	11:49	0.00	0.01	**0.10**	0.75	5.8	**4.2**	**4.0**	**1.8**	**-**	**0.1**	**-**
4	Nov 08	11:15			0.00			0.0	0.0	0.0	0.0	0.0	0.0
5	Jan 22	11:48	0.73	2.42	**8.12**	27.60	96.1	**62.0**	**55.2**	**34.0** (16.0)	**26.5**	**12.0**	**7.5**
6	Jan 23	12:35	0.16	0.66	**2.80**	11.96	51.6	0.0	0.0	0.0	0.0	0.0	0.0
7	Jan 25	14:09	1.46	4.47	**12.94**	37.77	111.8	**70.0** (8.0)	**54.5** (12.9)	**26.2**	**17.0** (3.5)	**13.4** (2.5)	**5.2** (6.0)
8	Mar 22	12:00	1.93	5.91	**18.11**	55.64	172.0	**21.3** (2.0)	**14.0** (1.7)	**7.5** (0.5)	**2.5** (0.4)	**0.8** (0.2)	**0.2** (0.2)

The deployment number (num.), dates and the time of high water (HW, GMT) are given for each deployment, along with predicted (Bayonet GPE) and measured (WireWall) total overtopping volumes (m^3^ m^−1^). Bold font indicates the mean, non-zero values for ease of comparison. For Bayonet GPE the total volume −2, −1, +1 and +2 s.d. values are given as well as the mean estimate. For WireWall the total volumes are given at various distances inland, i.e. from the different sensors located either at the crest (sensor 2), at the railing (sensor 3, 35 cm from the crest) or further inland (sensors 4, 5 and 6, located 70, 105 and 140 cm from the crest). An alternative, “sensible” volume for sensor 2 is also given, i.e. only those events that were also detected on sensor 3. Where more than one WireWall unit was working, the + /− range of values is shown by the numbers in brackets. Notes: Deployment 3 - only WireWall unit C was working, and sensors 4 and 6 were damaged; Deployment 5 - on the 22nd January only WireWall unit A was working, and the value from sensor 3 may be very uncertain; Deployment 7 - all sensors on the unit A worked, and most sensors on both the other two units (but not 3 and 5 on unit B, and not 3 and 6 on unit C). Deployment 8 - all sensors on both units A and C worked, but unit B failed.

The decrease in measured overtopping volumes with distance inland can be seen in Fig. 6. Water impacting the seawall is deflected upwards and some falls straight back into the sea, with only part of the plume of green water and/or dense spray being carried inland (sometimes aided by an on-shore wind). Sensor 1 projects seawards beyond the crest, and sensor 2 is at the crest hence much of the water seen on these sensors returns straight back into the sea. As the rest moves inland it falls to the ground and spreads out in all directions before returning to the sea aided by the slight slope of the promenade. The amount of water reaching the inland sensors depends on the speed and height of the plume, which determines the distance inland that the water can travel. A video clip showing the event in Fig. 6 is available in Supplementary Movie 1 (Movie_of_event_in_Figure_6.mov): other video clips are available on the internet^24^.

**Fig. 7:**
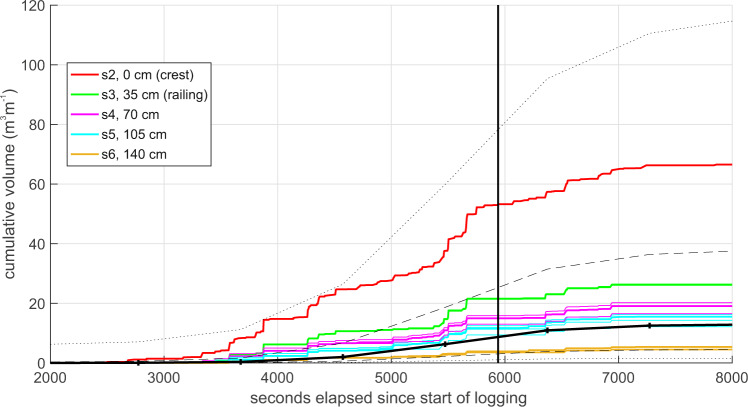
Cumulative overtopping measured by WireWall unit A on the 25th January 2019. Data were obtained at various distances inland from the crest as given in the key: sensor s2 at the crest (red line); sensor s3 at 35 cm inland (green line) next to the railing; sensor s4 at 70 cm inland (pink line); sensor s5 105 cm inland (pale blue line); sensor s6 at 140 cm inland (yellow line). All sensor pairs are shown for the WireWall data, e.g. overtopping at the railing is calculated from pairs 13 and 23 (results from both pairs are in green but overlie each other). No data are shown for sensor 1 since this was seawards of the crest. The vertical line indicates the time of high water. The thick black line is the Bayonet GPE prediction, dashed and dotted black lines are the Bayonet GPE + /−1 and 2 s.d. uncertainty estimates respectively. These data are available from the British Oceanographic Data Centre (10.5285/acd939f0-38e7-57b0-e053-6c86abc0aa19/).

The decrease in overtopping volumes with distance inland can also be seen in Fig. 7, which shows the cumulative volumes from the 6 sensors on unit A for the whole period during which overtopping occurred on the 25th January. Measurements from all sensor pairs are shown, as are the Bayonet GPE predictions of overtopping at the crest^25^. The mean Bayonet GPE volume underestimates the measured volume at the railing by about a factor of two, and is closer to the volumes measured about 1 m inland of the crest. However, the + /−2 s.d. uncertainty in the Bayonet GPE predictions is large enough to encompass all the WireWall data, from the crest to 1.4 m inland. The WireWall data show that, instead of the overtopping being symmetrical about the time of high water as might be expected, about 75% of the overtopping occurred before high water. This asymmetry was also observed in the other deployments and did not appear to be caused by changes in e.g. offshore wave conditions or wind speed or direction, but may be linked to changes in tidal currents that impact the nearshore wave field. Data from a nearby Acoustic Wave And Current system deployed at the low water mark showed a flood-dominance in the tidal currents. The impact of such forcing conditions on the measured overtopping is the subject of future work.

**Fig. 8:**
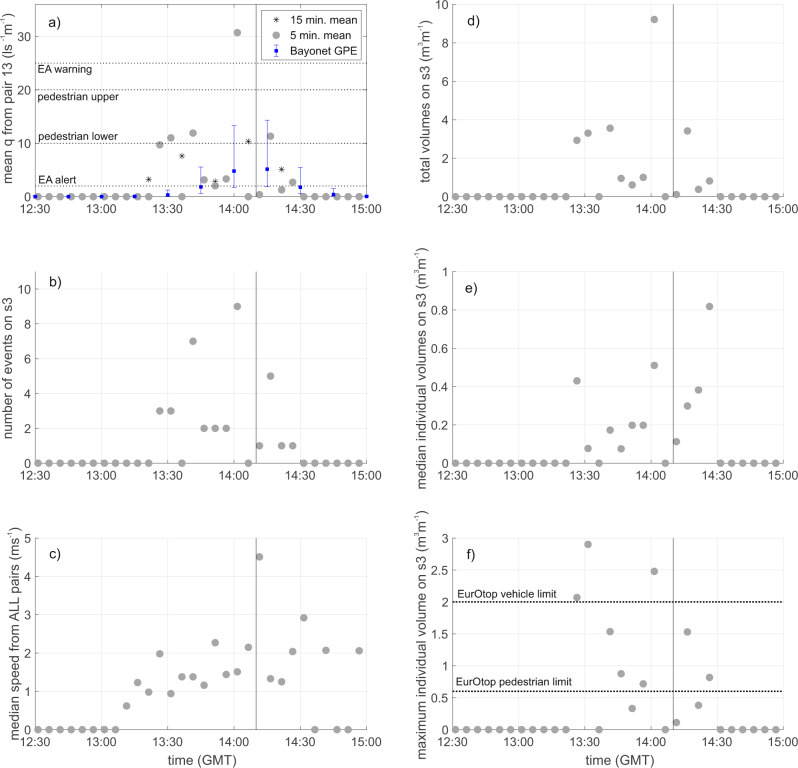
Time series of overtopping statistics for the deployment on 25th January 2019. **a**) mean discharge rates (l s^−1^ m^−1^) from the WireWall sensor 3 (s3) at the railing, calculated over 5 min (grey circles) and 15 min (black stars) intervals. Bayonet GPE 15 min predictions of overtopping at the crest (blue squares) are also shown with the error bars indicating + /−1 s.d. uncertainty; **b**) number of events detected at the railing per 5-minute interval; **c**) the median speed of the events from all sensor pairs. Simple statistics for volumes (m^3^ m^−1^) measured by sensor 3 at the railing are shown in the following panels: **d**) total volumes in each 5-minute interval; **e**) median of the individual wave volumes at the railing; **f**) the maximum individual wave volume at the railing. Horizontal dotted lines indicate various thresholds for EA (Environment Agency) alerts or warnings. Vertical lines indicate the time (GMT Greenwich Mean Time) of high water.

Since WireWall measures the speed and volume of individual waves, rather than just the total volume per deployment, the nature of the overtopping can be examined in more detail than is possible with traditional collection tanks or industry-standard tools such as Bayonet GPE. Figure 8 shows the data for the 25th January, broken down into separate 5 minute periods, for events that reached sensor 3, just inland of the handrail. The very sporadic nature of coastal overtopping is clearly seen: even near the time of high water the number of individual events seen in adjacent 5 minute periods varied from zero to 10. The median of the speeds in each 5 minute period was usually about 2 m s^−1^, except for one period where the single event detected on sensor 3 had a speed of 4.5 m s^−1^. Similarly, near the time of high water (HW) the total volumes in any 5 minute period varied from less than 1 m^3^ m^−1^ to over 9 m^3^ m^−1^. The maximum volume of any single event in a 5 minute period was even more variable, with the largest single volume of nearly 3 m^3^ m^−1^ being seen about 40 minutes before HW, not long after the start of overtopping. This maximum volume well exceeds the threshold specified as a hazard to vehicles (2 m^3^ m^−1^ for a single wave^1^) even though the mean discharge rate at the time was only 11 l s^−1^ m^−1^, just above the discharge limit for pedestrians.

**Fig. 9:**
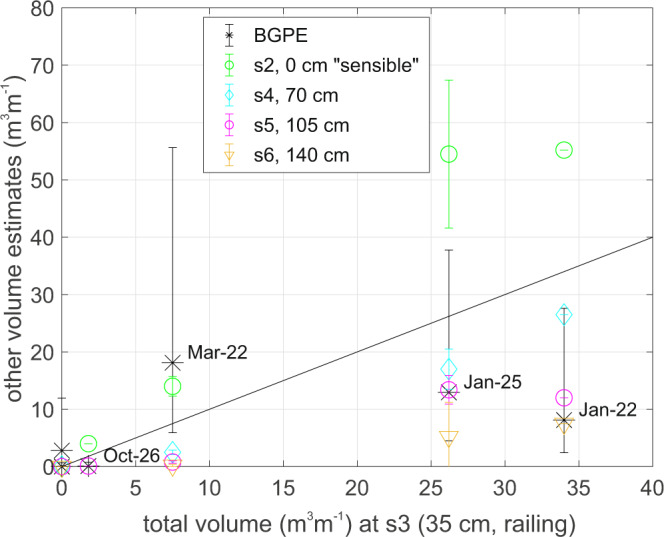
Total volumes for the various WireWall deployments at Crosby in winter 2018/2019. Deployment dates are shown, except for the two deployments where WireWall measured zero overtopping on the 25th October 2018 and 23rd January 2019. The *x*-axis shows the WireWall measurements for volumes measured at sensor 3 (s3) at the handrail (35 cm inland of the crest). The *y*-axis shows the WireWall measurements at the crest and at various distances inland of the crest; sensor s2 at the crest (green) but only using data from events that were detected at the railing too; sensor s4, 70 cm inland (pale blue); sensor 52, 105 cm inland (pink); sensor s6, 140 cm inland (yellow). Error bars show the range of WireWall values if more than one unit was working. Predictions from Bayonet GPE for overtopping at the crest (black stars) are also shown, with error bars indicating + /−1 s.d. uncertainty. The black line shows 1:1 agreement.

Figure 9 and Table 1 summarise the total volume of overtopping measured, or predicted, for each of the eight deployments. Overtopping was measured by WireWall during four of the eight deployments only: the presence or absence of overtopping was verified by video camera and by observation by the authors who were on site. Since much of the water detected by sensor 2 at the crest was seen to return directly into the sea, a second (sensible) estimate was calculated for sensor 2, which only included those events which were also detected on sensor 3, i.e. at least some of the water from those events did reach the handrail. Bayonet GPE predicted overtopping for six of the eight deployments. Compared to the WireWall results at the railing, Bayonet GPE predictions of overtopping at the crest under-predicted the total volumes for three out of four deployments where overtopping was measured, and over-predicted for the remaining one. However, for the three deployments with the largest overtopping volumes, the results from WireWall and the Bayonet GPE predictions are in reasonable agreement when the extent of the uncertainties in both are considered. Bayonet GPE predicted small amounts of overtopping (total volumes of 3 m^3^ m^−1^ or less, Table 1) on two of the deployments where none actually occurred.

## Discussion

Due to a dearth of data obtained in the field, the tools commonly used to predict overtopping are largely based on data from idealised flume studies and the predictions of total overtopping volumes and discharge rates from such tools have orders of magnitude uncertainty. The novel WireWall system was designed to address the lack of field data by providing wave-by-wave overtopping measurements of speeds and volumes. WireWall was successfully validated against traditional methods in flume tests: the WireWall results agreed closely with those obtained from collection tanks, and were in much better agreement than the predictions from Bayonet GPE. A number of short (~6 h) trial deployments at a sea wall in Crosby on the northwest coast of the UK demonstrated WireWall’s capability to obtain overtopping data in the field. The comparison of the WireWall field results with predictions from Bayonet GPE was similar to the flume comparison in that the very large (+/−1 s.d. of one to two orders of magnitude) uncertainty estimates in the Bayonet GPE predictions encompassed the WireWall results for the three field deployments during which the largest overtopping volumes occurred. For the deployment where more modest overtopping volumes (about 4 m^3^ m^−1^ total at the crest) were measured, the WireWall results were close to the +2 s.d. uncertainty estimate of the Bayonet GPE prediction. Bayonet GPE predicted modest overtopping during two deployments when none was observed. The less good performance of the Bayonet GPE predictions in the low-energy conditions is to be expected since the database underlying the tool is focussed on high-energy, hazardous conditions. In addition, the profile of the structure at Crosby was relatively unusual in that is was made up of a recurved wall fronted by a stepped revetment, and such structures are not well represented in EurOtop.

The wave-by-wave measurements from WireWall allow the nature of the overtopping to be investigated in detail. Initial observations show that at Crosby typical, i.e. commonly-occurring, winter conditions can pose a hazard to pedestrians and vehicles. Such high probability coastal conditions are out of range of the datasets used to develop hazard forecast tools, since they generally focus on low probability, extreme coastal conditions. Hence forecasts of these nuisance overtopping conditions contain very large uncertainties which impact hazard alert/warning capabilities. In addition the sporadic nature of overtopping means that wave-by-wave overtopping estimates may provide a better indication of hazard than more traditional measures such as mean discharge rates. For example, the largest individual wave volume measured at the handrail well exceeded the hazard limit for vehicles despite the mean discharge rate across the hour-long duration of overtopping being below the lower limit for hazard to pedestrians. Such detailed, accurate hazard information could be extremely valuable for the planning of operational services (e.g. at Crosby the RNLI emergency rescue services patrol the seafront in a small truck or quad bike and have their station positioned on the sea wall).

The WireWall data from the preliminary trial at Crosby, although limited in duration, has already produced some other interesting results. For example, the overtopping measured during the deployments at Crosby was not symmetrical about the time of high water, with about 3/4 of overtopping occurring on the rising tide. In future, such information will allow the forcing factors that drive overtopping to be better understood, and better prediction methods to be developed to improve forecasting of hazards and flooding. Similarly, the spatial arrangement of sensors within the WireWall system can be adapted to suit the data requirements: at Crosby the WireWall units each carried six sensors, partly for redundancy, but the distribution of the sensors was also designed so that the sea-land distribution of overtopping volumes could be investigated. Use of multiple sensors or units distributed along the sea wall would allow the along-shore variation of overtopping to be examined.

Our aim is to develop a system that is low-cost, robust, capable of being deployed in a wide range of locations, and provides ample redundancy against data loss due to damage. However, the system that was deployed at Crosby was a prototype and had various limitations. (1) The 2.4 m height of the sensors limited the size of overtopping plumes that can be measured. However, during the Crosby deployments the plumes rarely exceeded the height of the sensors. (2) Although the sensors rarely suffered damage during the field deployments themselves (despite pebbles and brick fragments being occasionally thrown on land by the overtopping) they did sometimes suffer damage when being mounted on the rig or during transportation. (3) The deployments were limited in duration, partly by the need for staff to be on site to assemble the system and then download and analyse data prior to the next deployment. (4) Data was sometimes lost due to water ingress in the connectors.

The limitations described above have largely been addressed by subsequent system development. Longer (4 m) sensors have been produced that are more robust while still being low-cost, and the electronics have been improved. A major advance has been the addition of on-board processing to the data logger. This allows housekeeping information and simple overtopping data (wave-by-wave overtopping frequencies and depths) to be telemetered in near-real time. In future, this could allow real-time overtopping hazard monitoring and provision of overtopping data for assimilation into hazard forecasting services and/or emerging observation-based nowcast services. This more robust system with real-time data telemetry has recently been deployed for 12 months on the south coast of the UK. The results are being analysed and will be the subject of future papers.

## Methods

### Principles of the WireWall measurement system

The WireWall system consists of a custom-designed, low power electronics unit which can record data from up to 6 capacitance sensors simultaneously. The electronics unit consists of a 32 bit ARM microcontroller along with a PCAP02 capacitance measuring integrated circuit, a real time clock with battery backup, flash memory and a memory card. The sensors are made up of two wires: (1) a silver plated copper wire (to form one constant area plate of a parallel plate capacitor) coated in PTFE (acting as the capacitor’s dielectric of constant thickness) and (2) an uninsulated tinned copper wire to provide a return circuit. The two are separated by a gap of about 1 cm. When water bridges this gap it acts as an electrically connected second plate of the capacitor. The capacitance changes linearly in relation to the wetted length of the capacitance wire. There is no information as to the location of the water on the wire: for example, ten separate 1 cm drops of water produce the same signal as a single 10 cm body of water. The capacitance of each sensor is sampled at 400 Hz, data are processed at that rate and data from all sensors on a given unit are time-stamped before storing on a local memory card. Data between units were also time-synchronised at this 400 Hz level by connecting them together with cabling which allows a synchronising signal (sample number) to be sent between them. The sensors were calibrated simply by immersing them in various known depths of water and applying a linear fit to the resulting capacitance signals. When deployed, the coax cables connecting the sensors to the logging unit added a constant offset to the output signal: this offset was up to about 10 cm (e.g. Fig. 6), depending on the cable length, and was removed automatically as part of the data processing.

In all the deployments (balloon, flume, and field tests) described here the sensors on a given unit were arranged in a row aligned with the expected direction of travel of the water. The time delay between the water arriving at different sensors allows the horizontal velocity of the water to be calculated. If the speed of the water is assumed to be constant (a reasonable assumption over short distances) then the volume (per linear meter) of water passing a sensor is given by:

1 $${{{{{\rm{volume}}}}}}({{{{{{\rm{m}}}}}}}^{3}{{{{{{\rm{m}}}}}}}^{-1})={{{{{\rm{speed}}}}}}({{{{{\rm{m}}}}}}\,{{{{{{\rm{s}}}}}}}^{-1})\ast {{{{{\rm{mean}}}}}}\,{{{{{\rm{wetted}}}}}}\,{{{{{\rm{depth}}}}}}({{{{{\rm{m}}}}}})\ast {{{{{\rm{duration}}}}}}\,{{{{{\rm{of}}}}}}\,{{{{{\rm{event}}}}}}\,({{{{{\rm{s}}}}}})$$

This depends on accurately determining the time of arrival of water at each sensor, and also determining the end of the event (i.e. when the water for an individual wave has passed the sensor) to obtain the event duration. In the flume the start of an event was defined as the time at which the measured depth increased rapidly, i.e. the rate of change of depth was above some threshold. The end of the event was determined as the time at which the measured depth returned close to the value recorded just before the start of the event (the baseline, or dry depth). In the field, this method could not be used due to pools of water that may have collected on the promenade: instead, the end of the event was determined from the variance of the signal falling below some threshold (the variance was much higher during an event - see Fig. 6). The mean depth is calculated as the measured minus baseline value (thus removing any offset) averaged over the duration of the event. The various thresholds were set by examining one or two runs (in the flume), or an hour or so of overtopping (in the field) in detail, i.e. visually checking the time series of wetted lengths and the resulting volumes to ensure that all events visible in the former are associated with a volume. Video analysis and visual observations by staff were also used to check that the events seen during that run/partial deployment were detected by the data processing. If the threshold was set too high then events would be missed and return zero volume. Conversely, if the threshold was set too low then any noise in the signal (e.g. small spray droplets arriving ahead of the main body of overtopping water) could be interpreted as a (false) event start and result in erroneously small speeds. The thresholds used in the field were different to those used in the flume since the magnitude of the events were a great deal larger. In the flume, the thresholds were set to capture very small events, with the smallest measured volume being of order 0.1 l m^−1^ from an event that had a mean wetted length of less than 1 cm and a duration of only 0.1 seconds (Eq. 1). In the field the start-of-event threshold was relaxed since random spray droplets of similar small size caused false starts to be detected. Once the thresholds were set they were almost always applied unchanged to the rest of the flume/field data. The exception was the first field deployment where overtopping occurred: this was on the 26th October 2018 and the total volume for the whole of that tide was only 2 m^3^ m^−1^. The thresholds used for this deployment were too low for the later deployments where total overtopping volumes were roughly an order of magnitude larger and a different threshold was used for those. In future, we will investigate setting the thresholds automatically, depending on the behaviour of the signals during dry periods compared to those during the events.

The volume calculation also depends on the accuracy of the measured speed which in turn depends on the distance between sensor pairs and the speed of the water. For example, if the sensor pair are separated by 10 cm (similar to the flume and balloon tests), and the water is travelling at 10 m s^−1^, then an event would take only 0.01 s, or 4 samples (at 400 Hz) to travel from one sensor to the next: errors of + /−1 sample would produce measured speeds of 12.5/7.5 m s^−1^, i.e. a volume error of + /−25%. However, in the flume the speeds were usually about 2 m s^−1^, so the uncertainty in the calculated velocity was about 5%. In the field the sensors were spaced 30 to 35 cm apart, and the maximum measured speeds were about 5 m s^−1^. Multiple estimates of the speed of the water were made for events where the water passed more than two sensors in the row. For example, the larger field events reached the most inland sensor (number 6 in the row), which allowed estimates to be made from all 15 permutations of sensors pairs. Where multiple estimates were available for a single event, the median of those estimates was applied to the depths from each sensor. Again this assumes that the speed of the flow was constant for each event, i.e. did not change as the overtopping water travelled inland. This is believed to be reasonable over small distances (sensor 6 was 1.4 m inland of the crest) but in practice, the speed of the water may decrease slightly as it travels through the air, or possibly increase if driven by a strong on-shore wind.

### Flume validation

The prototype WireWall system was validated in a wave flume at HR Wallingford prior to the field trials at Crosby. The flume was 45 m long, 1.2 m wide and 2 m deep and was equipped at one end with a piston-type wave paddle controlled by HR Wallingford’s Merlin software. At the other end was a 1:7.5 scale model of the Crosby sea defence (the northern end, where WireWall was to be deployed during the later field trials). The structure comprises of a vertical wall with a recurve that has a crest level of 6.4 m ODN (Ordnance Datum Newlyn) fronted by a stepped revetment. The bathymetry in the flume was formed to represent the low-gradient, wide sandy beach at Crosby as surveyed on the 11th December 2018. Wave spectra were generated to represent conditions that may be expected at Crosby using known wave conditions from an offshore wave buoy. The scaled up (real-world) conditions simulated in the tests presented here are given in Table 2, along with the real-world results from the long (~1000 wave) runs.

**Table 2:** Results from the flume tests used to compare overtopping discharge rate as measured by WireWall and predicted by Bayonet GPE.

				Tanks	T	WireWall	W	Bayonet GPE		
Test	WL m ODN	Hm0,t m	Tp,t s	q, l s^−1^ m^−1^	no.	q, l s^−1^ m^−1^	no.	q l s^−1^ m^−1^	−1 s.d.	+1 s.d.
WC01**	5.87	0.87	6.27	14.2 ± 2.1	5	14.0 ± 1.4	2	13.4	4.0	148
WC06	6.17	0.91	5.72	27.2 ± 2.3	2	-		71.8	14.4	1794
WC07	6.17	0.94	6.6	34.1 ± 4.5	4	28.3 ± 3.8	2	96.1	16.2	3382
WC12	4.98	0.87	6.27	0.4	1	-		0.3	0.1	6
WC13	5.33	0.87	6.27	1.5	1	-		0.5	0.1	10
WC14	5.80	0.83	6.42	9.1 ± 0.3	2	-		7.6	2.1	101
WC15**	5.62	0.8	7.65	8.4 ± 0.8	6	9.1 ± 1.6	4	3.1	0.6	89

The water levels (WL), significant wave heights (Hm0,t), wave peak periods (Tp,t) and discharge rates (q) are real-world values, i.e. scaled up from the flume values. Wave heights and periods are those at the toe of the structure, as indicated by “t” in the parameter names. The number of repeat runs for which tank (T no.) and/or WireWall (W no.) data were collected are provided. Tank data are the mean of all data available (from the HRW partitioned tank and/or one or more of the three large NOC tanks). WireWall values are averages from both units. Mean and + /− s.d. are given for discharge rates.

** indicates conditions that were also simulated when doing many short (~10 min) incomplete runs to avoid the use of pumps.

**Fig. 10:**
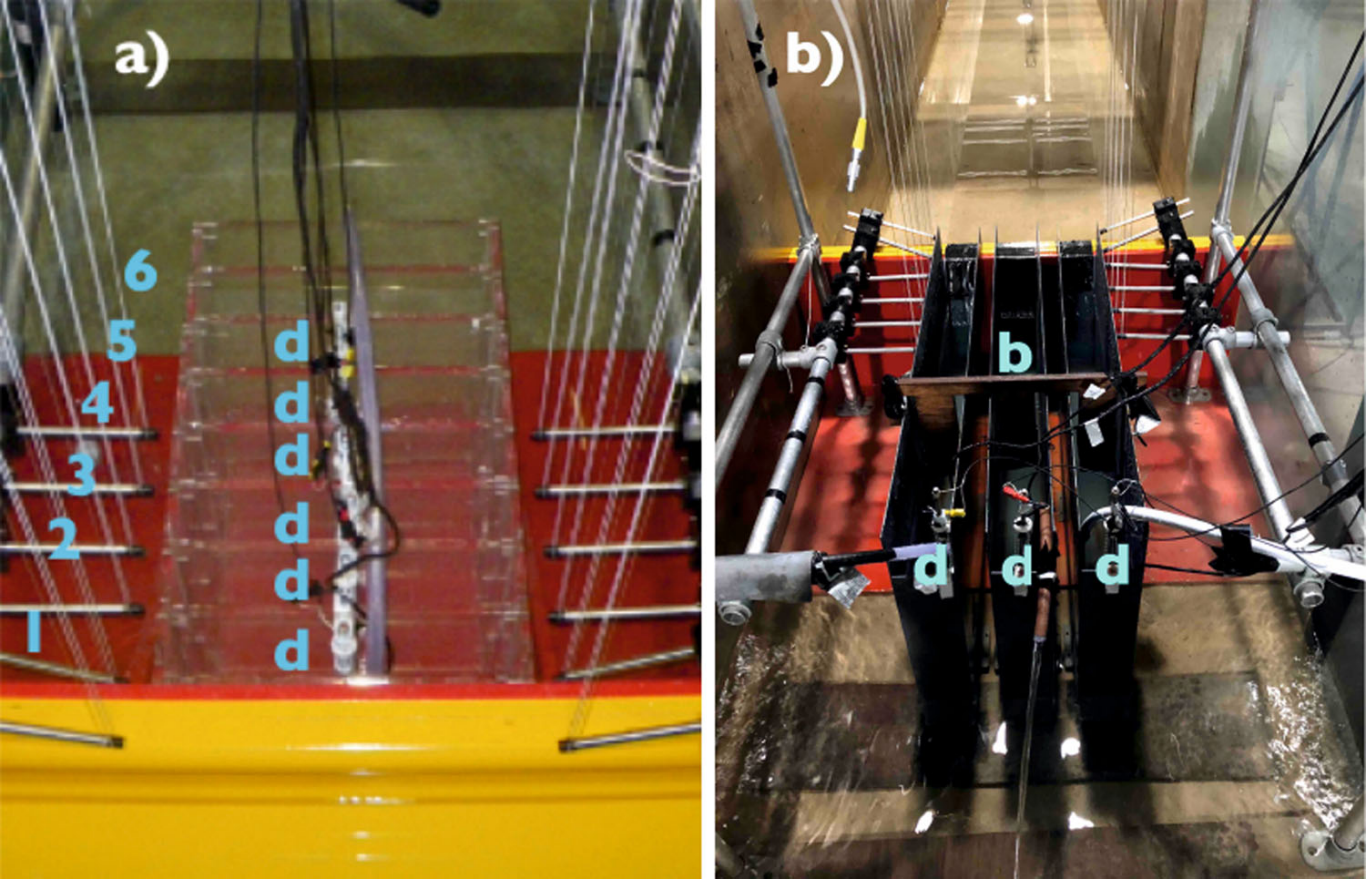
The experimental setup during the flume tests. **a**) The partitioned perspex collection tank used for the first flume tests (viewed looking inland). The six sensors on the two WireWall units are located to either side of the tank, arranged in rows oriented from the crest (sensor labelled “1” in blue) inland to sensor 6 (“6” in blue). The cables in the centre are attached to 6 other WireWall sensors (dipsticks, indicated by “d” in blue) which made continuous 1 Hz measurements of the water depth in the 6 partitions closet to the structure. **b**) The three much larger longitudinal tanks used for the later tests described in this paper (viewed looking offshore). Dipsticks (labelled “d” in blue) and pumps were located at the inland end of each of the three tanks. A wooden baffle (labelled “b” in blue) was used to damp the sloshing of the water in the tanks and the sides of the tanks were raised to prevent water splashing sideways from one tank to the next.

A measurement rig (1.2 m long, 0.8 m wide and 1.8 m high) to carry the WireWall sensors was built to fit within the flume. Two WireWall units were used, each with 6 sensors arranged in a row inland from the crest of the model sea defence, with the lower end of the sensor at the same height as the crest. Collection tanks were placed immediately inland of the model (Fig. 10), with the top of the tanks level with the crest. The perspex tank used during the first flume tests (Fig. 10a) was partitioned into 8 sections in an attempt to obtained data on the inland distribution of overtopping water, and the WireWall sensors were spaced a little under 8 cm apart to correspond to the tank partitions. The WireWall sensors on the two units were offset to either side of the tank, in order not to interfere with the flow of water into the tank. The tank was replaced in later tests by three much larger tanks which were arranged side by side (Fig. 10b), again with the WireWall sensors positioned to either side. For these tests the first sensor in each unit was mounted at the crest of the wall; the second just inland of the wall above the seawards-most edge of the tanks, 7 cm inland of the first sensor; the remaining four sensors were spaced 10 cm apart progressively further inland. Even though those tanks were much larger, pumps had to be used during the longer runs to prevent the tanks overflowing. In all cases the partitions/tanks held WireWall dipstick sensors that measured water depth continuously at 1 Hz.

Overtopping of a structure in a flume is assumed to be uniform across the width of the flume, but in order to avoid any non-uniformity close to the walls of the flume measurements are usually made in the middle. However, the WireWall sensors were located to either side of the collection tanks in order to minimise interference with the flow of water into the tanks. The use of three longitudinal tanks allowed us to investigate any cross-flume variation in overtopping that might affect the data from the WireWall units.

Due to the sporadic nature of overtopping, long flume runs of at least 1000 waves (~1 h in this study) are traditionally used to obtain total overtopping volumes for a given beach/structure profile. These data are then included in the EurOtop database on which the numerical tool Bayonet GPE is based. However, even the large longitudinal tanks began to overflow before such long runs could be completed, so pumps were inserted into the tanks to prevent them overflowing. The rate of flow through the pumps could not be changed and sometimes they pumped the tanks dry, so the pumps needed to be turned on and off a number of times during each of the long runs. This was done manually with the times that the pumps were switched on/off noted. Corrections were made based on the measured flow rate of the pumps but this introduced additional errors into the results for to various reasons. For example; pumps may be turned on late and some water may have already flowed out of the tank, particularly if a large wave event caused a lot of sloshing in the tanks; pumps may have been turned off late meaning they drew in air and did not restart for an unknown length of time after being turned back on; the times of switching on/off may be inaccurate.

To avoid large errors in the tank data, some of the WireWall validation tests were done using much shorter, incomplete runs of about 10 min duration. As well as avoiding the use of pumps in the collections tanks, these runs were short enough to be repeated many times in order to investigate the repeatability of the results.

### Field trials and comparison with Bayonet GPE predictions

**Fig. 11:**
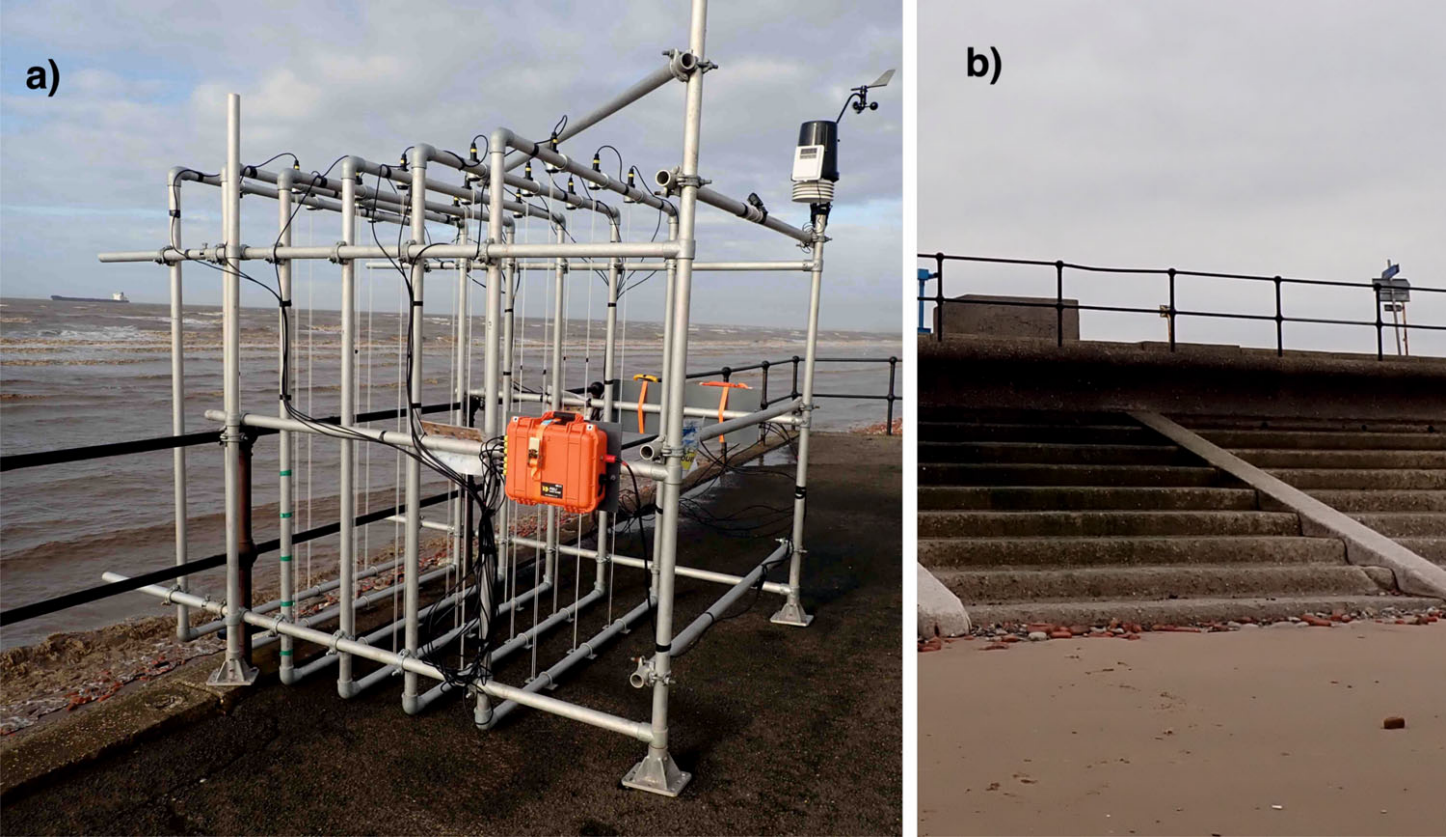
Images from the field deployment at Crosby. **a**) The WireWall system. **b**) The stepped revetment and the recurved wall structure.

The first trial deployments of the prototype WireWall system took place at Crosby, North West England, during winter 2018/2019. At this site a wide pedestrian walkway (promenade) runs along the top of the sea wall: the promendae slopes up gradually from a height of approximately 6.4 m ODN at the crest to 7.2 m ODN further inland at a splash wall. The vertical sea wall has a recurve at the top and a stepped revetment at its base (Fig. 11b). The sandy beach is wide and shallow with quantities of eroded house bricks near the toe of the structure. When choosing the exact deployment location consideration was given to access and safety as well as making sure the location was representative of the site in general. The location chosen was near the northern end of the defence where the promenade was wide enough that the rig did not obstruct the public or other users and was close to an access gate. The site was vulnerable to wave overtopping, but unaffected by the local influence of smaller scale features (e.g. the slipway further south). This site was just north of the Hall Road beach profile which was used in the numerical overtopping predictions: the data are therefore suitable for comparison with the numerical predictions presented here.

The WireWall system was installed temporarily for eight tides, either for the duration of a single tidal cycle or for two or three days, when tides exceeded MHWS and the wave and weather forecasts suggested that overtopping might occur^24^. Data were collected during the daytime tides when NOC staff were present to operate the electronics and video cameras (both were removed overnight) and make visual observations of the conditions. Prior to each field deployment the sensors were calibrated at the NOC lab in Liverpool. Post-deployment the sensors were tested for any damage, e.g. to the PTFE coating of the capacitance wires. The sensor calibrations were also checked post-deployment (for un-damaged sensors) and were unchanged.

The system deployed at Crosby was only a little different from that used in the flume. It used exactly the same electronics units but the sensors were made of thicker wires (to be more robust against damage) and were 2.4 m tall. Similar cheap, readily available materials (aluminium scaffold poles and key clamps) were used to build the rig, which was considerably larger than the one used in the flume. Use of such materials allows the rig to be easily re-configured to suit a wide range of possible deployment sites in the future. The rig was clamped to the hand rail on the sea wall, and the four feet of the rig were bolted into the surface of the walkway. The sensors were attached to the rig using a tensioning system at the upper end. At the lower end the uninsulated wire was terminated and the PTFE coated wire was looped through a bespoke fitting that allowed water to run off rather than pool. The PTFE wire was looped back up and terminated in the connector at the top (doubling the wire in this way doubled its the sensitivity). The bespoke fitting was also used to protect the lower end of the capacitance wire from the ground while assembling the system, and from any debris that may have washed along the promenade: unfortunately the sharp edges on some of the fittings tended to damage the PTFE coating when the wires were being threaded, and were initially a cause of data loss.

The rig carried three WireWall units, all time-synchronized. Each unit logged the 400 Hz data from six sensors arranged in a row oriented from sea to land. The seawards-most sensor (number 1) projected seawards of the crest by about 35 cm, sensor 2 was at the crest, sensor 3 a little inland of the handrail, and the others were spaced 35 cm progressively further in land. There was room within the rig to increase sensor spacing if desired but this did not prove necessary. The three rows of sensors were separated in the along-shore direction by about 45 cm from each other. The use of multiple units was designed to allow us to assess spatial variability and also to provide additional redundancy in case of damage.

Before and after each WireWall deployment profiles of the beach were collected using equipment borrowed from Sefton Council, and performed in the same fashion so that the data could be included in the Regional Monitoring Programme. Additional profile data were collected at toe and the stepped revetment of the infrastructure. Data from the local WaveNet buoy, UK tide gauge and the nearby UK Met Office weather station were obtained for each deployment. These data provided input forcing to the numerical model (SWAN, Simulating WAves Nearshore^28^) used to transform offshore wave conditions using nearshore water levels to the toe of the sea wall. Conditions at the toe were then input to Bayonet GPE to produce the predictions of overtopping volumes and discharge rates presented here. After the first deployment during which overtopping was detected (26th October, 2018) comparisons of the Bayonet GPE predictions with the WireWall measurements showed a discrepancy of over two orders of magnitude. Further investigation indicated that this was due to an issue in the input parameters for Bayonet GPE: when these were corrected the comparison showed agreement to within the +2 s.d. uncertainty bound (5.8 m^3^ m^−1^ total volume) of the Bayonet GPE predictions. Bayonet GPE predictions of overtopping rates for subsequent deployments used the same (corrected) input parameters.

### Supplementary information

Description of Additional Supplementary Files

Supplementary Movie 1

## Data Availability

The datasets used in the current study are available in the British Oceanographic Data Centre (BODC) repository. The balloon, flume and field data can be access via the following links respectively: https://www.bodc.ac.uk/data/published_data_library/catalogue/10.5285/acd939f0-38e8-57b0-e053-6c86abc0aa19/ https://www.bodc.ac.uk/data/published_data_library/catalogue/10.5285/acd939f0-38e6-57b0-e053-6c86abc0aa19/ https://www.bodc.ac.uk/data/published_data_library/catalogue/10.5285/acd939f0-38e7-57b0-e053-6c86abc0aa19/.
