# Supplementary material for: A system for in-situ, wave-by-wave measurements of the speed and volume of coastal overtopping
Source: Commun Eng. 2023 Mar 3;2:9. doi: 10.1038/s44172-023-00058-3 (PMC10955827; doi:10.1038/s44172-023-00058-3)
Supplement: Supplementary file 1 — Description of Additional Supplementary Files [file 44172_2023_58_MOESM1_ESM.pdf]

Description of Additional Supplementary File

File name: Supplementary Movie 1

Description: A video clip showing the event in Figure 6.
